# Prevalence and prognostic significance of chronic respiratory diseases among hospitalized patients with COVID-19 infection: a single-center study

**DOI:** 10.1186/s43168-022-00172-4

**Published:** 2022-12-22

**Authors:** E. Abdelghany, Rasha A. Abdelfattah, S. Rabea Shehata, A. Abdelaziz

**Affiliations:** Department of Chest Diseases, Faculty of Medicine, Minia University, Minia, Egypt

**Keywords:** COVID-19 patients, Chronic respiratory disease (CRD)

## Abstract

**Background:**

WHO recognized the COVID-19 outbreak in China as a pandemic crisis on March 11, 2020. Patients with chronic respiratory diseases (CRDs) have limited physiological reserve; this lead to the assumption that COVID-19 infection in such patients could carry worse prognosis.

**Aim of study:**

To detect the prevalence and prognostic significance of CRDs among hospitalized patients with COVID-19 infection.

**Methods:**

The study was carried out at Minia Cardiothoracic University Hospital; all hospitalized COVID-19 patients during the period from January 2021 to August 2021 were included.

Patients were subjected to full medical history taking, full blood count, inflammatory markers (CRP, serum ferritin, serum lactate dehydrogenase (LDH), serum D-dimer, PCR for COVID-19 infection), and HRCT chest.

Need for and duration of mechanical ventilation whether invasive or non-invasive, duration of hospital stay, and condition at hospital discharge were recorded.

Diagnosis for chronic respiratory disease was considered when patients have documented previous history and investigations compatible with the diagnosis, e.g., previous pulmonary function tests, chest CT, or sleep study.

**Results:**

Comorbid chronic respiratory diseases were present in 57 patients (17.6%). Regarding presenting symptoms, no significant difference exists between patients with and without CRDs except for sputum production which was more frequent among patients with underlying CRDs.

Elevated inflammatory markers (ferritin, D-dimer, and LDH) were more frequently observed in patients without CRDs (*p* < 0.0001, 0.033, and 0.008, respectively).

COVID-19 with comorbid CRDs patients were more hypoxemic at presentation than other patients (*p* = 0.032).

There was significant number of COVID-19 patients with CRDs were discharged on home oxygen therapy (*p* = 0.003).

Regarding mortality in our cohort of patients, no significant difference exist between patients with and without CRDs (*p* 0.374)

Among patients with comorbid CRDs, the highest mortality was observed on patients with OSA followed by ILDS and then COPD.

**Conclusion:**

The presence of CRD was not found to be a poor prognostic value of COVID-19. Inflammatory markers (ferritin, D-dimer, and LDH) were significantly higher in COVID-19 patients without CRD than COVID-19 with CRD.

## Background

The World Health Organization (WHO) recognized the COVID-19 outbreak in China as a public health emergency of international concern on January 30, 2020 [1] and a pandemic crisis on March 11, 2020 [2].

Evidence that is rapidly developing shows that people with comorbidities have a significantly higher burden and infection rates than people without comorbidities [3].

Among the comorbidities, chronic respiratory disease (CRDs) has the third-highest fatality ratio after cardiovascular disease and diabetes [4].

Because of the limited physiological reserve in patients with CRDs, they thought to be at risk of developing severe forms of COVID-19 infection [5]. Indeed, COVID-19 causes a variety of respiratory symptoms, ranging from cough and dyspnea to the most severe form of acute respiratory distress syndrome [6].

There is an increasing concern that patients with prior CRDs may be at increased risk of getting severe infection and death due to COVID-19 infection.

The current study aimed at evaluation of the prevalence of CRDs in hospitalized COVID-19 patients and to evaluate their prognosis.

## Methods

The present study is a cross-sectional study that was carried out at Minia Cardiothoracic University Hospital, during the period from January 2021 to August 2021. The study included all admitted COVID-19 patients during the study period.

The nature of the present study was explained to all patients. The laboratory and radiological investigations are standard of care and posed no ethical conflicts. A consent was obtained for the patients himself or his next of kin. The study was approved by the hospital research ethics board of Minia University.

Diagnosis for chronic respiratory diseases was considered when patients have documented previous history and investigations compatible with the diagnosis, e.g., previous pulmonary function tests, chest CT, or sleep study. Patients were followed up until hospital discharge.

Patients were subjected to the following:

1. Thorough medical history, stressing on smoking history, onset, course and duration of symptoms, and associated comorbidities
2. Complete blood count with differential cell count
3. Inflammatory markers in the form of CRP, serum ferritin, serum lactate dehydrogenase (LDH), serum D-dimer
4. PCR test was done for all cases.
5. HRCT chest
6. Need for and duration of mechanical ventilation whether invasive or non-invasive were recorded.
7. Duration of hospitalization
8. Need for oxygen therapy at time of hospital discharge

### Statistical analysis

All data were entered into spreadsheet format in a statistical software program (IBM SPSS, version 20). Data collected through history taking, basic clinical examination, laboratory investigations, and outcome measures were coded, entered, and analyzed. According to the type of data, qualitative data were represented as number and percentage, and quantitative data were represented by mean ± SD. Independent sample *t*-test and ANOVA test were used for comparing means and correlation for association between two quantitative variables. Chi-square test is for categorical data. All statistical analyses were performed considering a *p*-value of < 0.05 as being statistically significant.

## Results

The current study involved 323 patients. They were classified into two groups: group 1 COVID-19 patients with chronic chest diseases included 57 (17.6%) patients and group 2 COVID-19 patients without chronic chest diseases 266 patients.

**Fig. 1:**
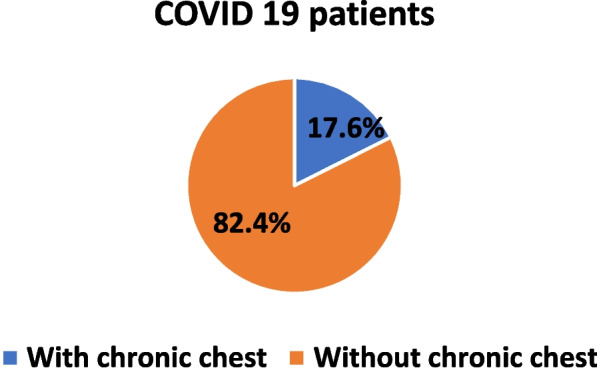
Prevalence of chronic respiratory diseases among COVID-19 patients. Fifty-seven patients had comorbid chronic respiratory disease

Figure 1 shows the prevalence of CRDs in the studied patients to be 17.6%.

Table 1 describes the prognostic features of COVID-19 patients according to the type of comorbid CRDs. Twenty-seven patients have COPD, 16 have OSA, 9 have ILD, 2 have asthma, and 1 has bronchiectasis; the highest mortality was observed on patients with OSA followed by ILDS and then COPD.

**Table 1:** Showing prognostic features of COVID-19 patients according to the type of comorbid chronic respiratory disease

	COPD ***N*** = 29	OSA ***N*** = 16	ILD ***N*** = 9	Asthma ***N*** = 2	Bronchiectasis ***N*** = 1
**Discharge Condition**					
**Dead**	6 (20.7%)	7 (43.8%)	2 (22.2%)	0	0
**On oxygen**	13 (44.8%)	8 (50%)	7 (77.8%)	0	1 (100%)
**Room air**	10 (34.5%)	1 (6.3%)	0	2 (100%)	0
**NIV**					
**Need**	13 (44.8%)	13 (81.3%)	3 (33.3%)	0	0
**No need**	16 (55.2%)	3 (18.8%)	6 (66.7%)	2 (100%)	1 (100%)
**IMV**					
**Need**	6 (20.7%)	5 (31.3%)	1 (11.1%)	0	0
**No need**	23 (79.3%)	11 (68.8%)	8 (88.9%)	2 (100%)	1 (100%)

*NIV* Noninvasive ventilation, *IMV* Invasive mechanical ventilation, *COPD* Chronic obstructive air way disease, *OSA* Obstructive sleep apnea, *ILD* Interstitial lung disease

The difference in clinical picture in COVID-19 patients with CRDs and without CRDs is described in Table 2, as there was no significant difference in symptoms between both groups except for sputum production which is more frequent in COVID-19 patients with CRDs (*p* = 0.002).

**Table 2:** Difference in clinical picture in COVID-19 patients with CRD and without CRD

***C***/***P***	COVID-19 patients with chronic chest disease ***N*** = 57	COVID-19 patients without chronic chest disease ***N*** = 266	***p-***value
	***N*** %	***N*** %	
**Fever**			
**Yes**	42 (73.7%)	198 (74.4%)	0.906
**Sore throat**			
**Yes**	28 (49.1%)	148 (55.6%)	0.370
**Cough**			
**Yes**	40 (70.2%)	161 (60.5%)	0.173
**Sputum production**			
**Yes**	35 (61.4%)	100 (37.5%)	0.002*
**Generalized bone pain**			
**Yes**	31 (54.4%)	168 (63.2%)	0.217
**Diarrhea**			
**Yes**	8 (14%)	42 (15.8%)	0.740
**Loss of taste & smell**			
**Yes**	2 (3.5%)	22 (8.3%)	0.214
**Abdominal pain**			
**Yes**	15 (26.3%)	87 (32.7%)	0.346

The mean RR is higher in COVID-19 patients without CRDs (*p* = 0.040), but COVID-19 with CRDs patients were more hypoxemic than others (*p* = 0.032) as presented in Table 3.

**Table 3:** Difference in vital signs between COVID-19 patients with CRD and patients without CRD

	COVID-19 patients with chronic chest disease ***N*** = 57	COVID-19 patients without chronic chest disease ***N*** = 266	***p***-value
	Mean ± SD	Mean ± SD	
**Temperature**	37.8 ± 0.6	37.7 ± 0.6	0.458
**Respiratory rate**	19 ± 4	21 ± 8	0.040*
**Heart rate**	81 ± 14	84 ± 17	0.120
**SPO2 at admission**	66 ± 16	74 ± 16	0.032*

On comparing laboratory investigations between the two groups of patients in Table 4, ferritin was significantly higher in COVID-19 patient without CRDs than patients with CRDs (*p* < 0.0001). Also D-dimer and serum LDH were significantly higher in COVID-19 patients without CRDs patients than patients with CRDs (*p* = 0.033 and *p* = 0.008, respectively).

**Table 4:** difference in laboratory investigations between the two groups

	COVID-19 patients with chronic chest ***N*** = 57	C0VID-19 patients without chronic chest *N* = 266	***p***-value
	***N*** %	***N*** %	
**TLC**			
**< 4000**	52 (91.2%)	242 (91.7%)	0.914
**• ≥ 4000**	5 (8.8%)	24 (9.02%)	
**Lymphocytes**			
**• < 1000**	34 (59.6%)	168 (63.1%)	0.42
**• ≥ 1000**	23 (40.4%)	98 (36.8%)	
**CRP**			
**• ≤ 10**	2 (3.5%)	8 (3.1%)	0.605
**• > 10**	55 (96.5%)	258 (96.9%)	
**D-dimer**			
**• ≤ 0.5**	23 (40.4%)	70 (26.3%)	0.033*
**• > 0.5**	34 (59.6%)	196 (73.7%)	
**Ferritin**			
**• ≤ 360**	27 (47.4%)	43 (16.1%)	< 0.0001*
**• > 360**	30 (52.6%)	223 (83.8%)	
**LDH**			
**• ≤ 280**	13 (23.6%)	30 (11.2%)	0.008*
**• > 280**	42 (76.4%)	236 (88.7%)	

*TLC* Total leucocyte count, lymph lymphocytes count, *CRP* C-reactive protein, *LDH* Lactate dehydrogenase

Table 5 shows difference in the prognostic features between the two groups. There was significant number of COVID-19 patients with CRDs were discharged on home oxygen therapy (*p* = 0.003).

**Table 5:** Showing difference in the prognostic features between the two groups

	COVID-19 patients with chronic chest disease( 57)	COVID-19 patients without chronic chest disease (266)	***p***-value
**Condition on discharge**	(*N* %)	(*N* %)	
**Dead**	15 (26.3%)	86 (32.3%)	0.374
**On oxygen**	29 (50.9%)	81 (30.5%)	0.003*
**Room air**	13 (22.8%)	99 (37.2%)	0.038*
**NIV**			
**Need**	29 (50.9%)	142 (53.4%)	0.731
**IMV**			
**Need**	12 (21.1%)	63 (23.7%)	0.669

*NIV* Noninvasive ventilation, *IMV* Invasive mechanical ventilation

Regarding COVID-19 infection severity, there is no significant difference between both groups as shown in Table 6.

**Table 6:** Distribution of disease severity among the two groups

Degree of severity	COVID-19 patients with chronic chest disease (57)	COVID-19 patients without chronic chest disease (266)	***p***-value
**Mod**	1 (1.8%)	16 (6.0%)	
**Severe**	27 (47.4%)	100 (37.6%)	0.886
**Critical**	29 (50.9%)	150 (56.4%)	

## Discussion

The prevalence of CRD in patients with COVID-19 infection seems to be lower than that in the general population; however, this should not viewed as a protective role of chronic CRD against COVID-19 infection [7].

In the present study, comorbid CRD were present in 57 (17.6%) of COVID-19 patients. Two previous studies compare the prevalence of CRD in patients hospitalized for COVID-19 with those who were hospitalized due to influenza; they demonstrated that COVID-19 patients were significantly less likely to have a history of CRD than in patients with influenza [8, 9].

Gülsen et al. [10] evaluated 14 studies that included 44,041 patients with COVID-19 infection; they found that CRD was present in 8.6% in patients with severe COVID-19 infection, while in patients with non-severe, CRD was present in 5.7%.

Lower prevalence of COPD (1.5%) in patients with COVID-19 was reported in a study [11] that included 1590 patients; it was found that out of 24 patients with COVID-19 and comorbid COPD 7 (29.2%) needed ICU admission, 5 (20.8%) required IMV, and death occurred in 5 (25%) patients. In the study conducted by Riou et al. [12], COPD was present in 15 (12%) of their studied patients which approached the prevalence of COPD in the general population in France; COPD prevalence was estimated between 1.0 and 11.1%.

In another study [10], COPD was present in 5.2% (2191/42,373) of patients with severe COVID-19 and in 1.4% (4203/306,151) of patients with non-severe COVID-19.

In the current study, 29 (8.98%) patients had COPD; 6 of them died in hospital (20.7%).

Knowledge from current literatures demonstrated the absence of significant association of asthma and increased risk or poor prognosis of COVID-19 infection [13], a finding that could seem surprising since it is well known that respiratory viral infections are major contributors to asthma exacerbation [14].

The prevalence of asthma in patients with COVID-19 infection showed marked variability in different studies. Based on 131 studies that included more than 400,000 patients [15], it was found that asthma prevalence in COVID-19 patients varies in different countries and regions ranging from 1.1 to 16.9%.

In a meta-analysis [10] that evaluated 18 studies on COVID-19 infection with comorbid asthma, the authors found asthma to be present in 2.3% (1873/81,319) of patients with severe COVID-19 and in 2.2% (11,796/538,737) of patients with non-severe COVID-19; data from China [16] showed a low prevalence of asthma among patients with COVID-19 and attributed this to a potential TH2-mediated protection from COVID-19 in patients with asthma. Asthma is not associated with higher COVID-19 severity or worse prognosis, and patients with asthma are found to have a lower risk of death compared with patients without asthma [15].

Only two (0.62%) patients of our cohort had underlying comorbid bronchial asthma, and both patients were discharged home safely.

In > 12,000 patients with asthma, it was found that asthma does not increased the risk of severe COVID-19 [10]. A US study compared COVID-19 patients with asthma to COVID-19 patients without asthma; the results of the study showed that the risk of hospitalization was similar in the two groups: nonsignificant difference regarding ICU admission in both groups and death markedly less likely in people with asthma and COVID-19 [17].

Data about the association between obstructive sleep apnea (OSA) and an increased risk of COVID-19 infection are lacking [18].

In the current study, 16 (4.95%) patients had OSA; the hospital mortality of patients with OSA was 7 (43.8%) patients.

Our results are in agreement with a previous study [19], which found that OSA is a risk factor for severe diseases, need for ICU and mechanical ventilation in patients with COVID-19 infection [20].

Emerging data indicate that interstitial lung disease is a poor prognostic factor in patients with COVID-19 infection especially when compared with other patients without ILD [21].

In our current study, 9 (2.8%) patients had ILDs; 2 (22.2%) patients died in hospital.

It was found that only 1% of patients with interstitial lung disease were hospitalized for COVID-19 among 401 patients in a single Belgian center [22].

The International Severe Acute Respiratory and Emerging Infection Consortium (ISARIC) evaluated the risk of death in COVID-19 patients with interstitial lung diseases; the study found that people with more severe restriction had higher mortality [23].

Only one patient in our study group had comorbid bronchiectasis, and patient improved. Among patients with cystic fibrosis, outcomes have not been as severe as initially expected with a global registry report of four (2.7%) deaths from 149 patients with SARS-CoV-2 infection [24].

Differentiating symptoms of COVID-19 infection from chronic underlying symptoms, or those of an acute COPD exacerbation may be challenging. If there is suspicion for COVID-19, testing for SARS-CoV-2 should be considered [7].

In the current study, regarding the symptoms, there was no significant difference in clinical manifestation among COVID-19 patients with CRD and COVID-19 patients without CRD except for sputum production which was more common in COVID-19 patients with chronic chest disease patients (*p* = 0.002).

A study found no significant differences in symptoms between COVID-19 patients with and without COPD, including fever, cough, and sputum production; however, patients with COPD were more likely to develop fatigue (56.0% vs. 40.2%), shortness of breath (66.0% vs. 26.3%), diarrhea (16.0% vs. 3.6%), and unconsciousness (8.0% vs. 1.7%) [25], by comparing COVID-19 patients with CRD with those without chronic underlying pulmonary diseases. Riou et al. [12] found that symptoms were similar in both groups except for fever which was significantly more common in patients without underlying lung disease. Crepitation was more frequently found in patients without chronic underlying LD (84% vs. 58%). Wheezy more frequent finding in patients with chronic pulmonary disease (18% vs. 2.7%).

Our results revealed that ferritin was significantly higher in COVID-19 patient without CRD than patients with CRD (*p* < 0.0001). Also D-dimer and serum LDH were significantly higher in COVID-19 patients without CRD patients than patients with CRD (*p* = 0.033 and *p* = 0.008, respectively).

Riou et al. [12] found that CRD were not a risk factor for ICU management. However, a tendency to higher global mortality was observed in COVID-19 patients with CRD, though this was not considered a risk factor for death in the multivariate logistic regression analysis. Similar results have been described in a recent meta-analysis [26]. However, data from two studies have revealed a higher mortality rate of 60% [27, 28].

In the current study, the need for mechanical ventilation whether noninvasive or invasive was not significantly different in both groups of patients (*p* = 0.731, 0.669 respectively). Also, there is no significant difference regarding hospital mortality in patients with COVID-19 and comorbid CRD, 15 (26.3%) when compared with patients without CRD, 86 (32.3%) (*p* = 0.347); however, 29 (50.9%) patients with comorbid COVID-19 and CRD need home oxygen therapy when discharged, and this was statistically higher than the group without CRD 81 (30.5%) (*p* = 0.003*).

Beltramo et al. [9] compared the occurrence of respiratory complications, need for ICU, and mortality in patients suffering from COVID-19 with and without CRD; they observed a significant increase in need for ICU admission and mortality in patients with CRD.

Wu et al. [25] found that in COPD patients, 12 patients (24%) need IMV compared to 49 (4.9) in patients without COPD (*p* 0.003), 20 patients (40%) with COPD needed NIV compared to 112 (11.2) in the non-COPD group (*p* 0.49), and duration of hospitalization was more in the COPD group, 11 days versus 10 (*p* = 0.05).

## Conclusion

The prevalence of comorbid CRD in our cohort of COVID-19 patients was 17.6%. The presence of CRD was not found to be a poor prognostic factor in our group of patients.

There was no significant difference regarding symptoms between COVID-19 patients with CRD and COVID-19 patients without CRD except for sputum production which was more frequent among patients with comorbid CRD.

Inflammatory markers (ferritin, D-dimer, and LDH) were significantly higher in COVID-19 patients without CRD.

The study is limited by the small number of patients who have underlying comorbid chronic respiratory disease.

## Data Availability

All data generated or analyzed during this study are included in this published article.

## Abbreviations

CRD: Chronic respiratory disease
NIV: Noninvasive ventilation
IMV: Invasive mechanical ventilation
COPD: Chronic obstructive airway disease
OSA: Obstructive sleep apnea
ILD: Interstitial lung disease
RR: Respiratory rate
HR: Heart rate
SpO2: Oxygen saturation
TLC: Total leucocyte count
Lymph: Lymphocytes count
CRP: C-reactive protein
LDH: Lactate dehydrogenase
